# Misdiagnosed desmoid fibromatosis of the chest wall presenting in emergency like as recurrence of post-traumatic hematoma: A case report and review of the literature

**DOI:** 10.1016/j.ijscr.2022.107019

**Published:** 2022-04-04

**Authors:** Giuseppe Evola, Mario Scravaglieri, Enrico Piazzese, Francesco Roberto Evola, Giovanni Francesco Di Fede, Luigi Piazza

**Affiliations:** a General and Emergency Surgery Department, Garibaldi Hospital, Piazza Santa Maria di Gesù 5, 95124 Catania, Italy; b Department of Orthopedic and Traumatology, Cannizzaro Hospital, via Messina 829, 95126 Catania, Italy; c Department of Diagnostic Radiology, Neuroradiology and Interventional Radiology, Garibaldi Hospital, Piazza Santa Maria di Gesù 5, 95124 Catania, Italy

**Keywords:** Desmoid fibromatosis, Chest wall, Systemic therapy, Surgery, Case report

## Abstract

**Introduction and importance:**

Desmoid Fibromatosis (DF) represents a rare neoplasm developing from fascial and musculoaponeurotic structures. Preoperative diagnosis of DF is a challenge because of its rarity and nonspecific presentation. Imaging may be helpful for determining the correct diagnosis. Currently there are different clinical treatments of DF including surgical treatment, drug treatment and radiotherapy.

**Case presentation:**

A 43-year-old Caucasian male presented to the Emergency Department with a 6-month history of recurrence of post-traumatic chest wall hematoma. Physical examination revealed a partially solid, painless mass on the right anterior chest wall. Laboratory tests reported and neutrophilic leukocytosis. Thoracic contrast-enhanced computed tomography showed a smooth contour, heterogeneous and hypodense subcutaneous soft tissue mass anterior to the right pectoral muscles and to the right 4th–7th rib. The patient underwent surgery: a solid suprafascial neoplasm was completely excised. The postoperative course of the patient was uneventful.

**Clinical discussion:**

DF is a soft tissue neoplasm with a tendency for local invasion and recurrence. The course of DF cannot be predicted, being fatal if DF infiltrates vital structures. Diagnosis of DF is difficult and imaging may be helpful for determining the correct diagnosis. Currently the treatment for DF has shifted from surgery (post-operative recurrence rates of 20%–70%) to conservative therapy including watchful waiting.

**Conclusion:**

DF is a myofibroblastic proliferative soft tissue tumor and classified as an intermediate malignancy. Preoperative diagnosis of DF needs a high index of suspicion and is facilitated by imaging. Surgery, among different treatments, represents a potentially curative treatment of DF.

## Highlights

• Desmoid Fibromatosis (DF) represents a rare neoplasm developing from fascial and musculoaponeurotic structures.
• The central biologic event in the DF formation is an alteration in the Wnt/β-catenin pathway.
• Diagnosis of DF is a challenge because of the absence of specific clinical presentation, pathognomonic radiographic and laboratory findings.
• Surgery, among different treatments, represents a potentially curative treatment of DF.

## 1 Introduction

Desmoid Fibromatosis (DF), also known as desmoid tumor, represents a rare neoplasm developing from fascial and musculoaponeurotic structures. DF accounts for approximately 0.03% of all neoplasms and less than 3% of all soft tissue tumors [1], with an annual incidence of 2–4/million [2]. DF can occur anywhere in the body and is characterized by slow progressive growth, local invasion and local recurrence after surgery but does not exhibit metastatic potential. Diagnosis needs a high index of suspicion and is based on clinical symptoms, detailed physical examination and radiology. The course of DF cannot be predicted, being fatal if DF infiltrates vital structures. Currently there are different clinical treatments of DF [3]. A case of DF of the chest wall, presenting in emergency like as recurrence of post-traumatic hematoma, is reported in accordance with SCARE 2020 criteria [4]. The purpose of this case report is to remember that diagnosis of DF is difficult and surgery represents a potentially curative treatment.

## 2 Presentation of case

**Fig. 1:**
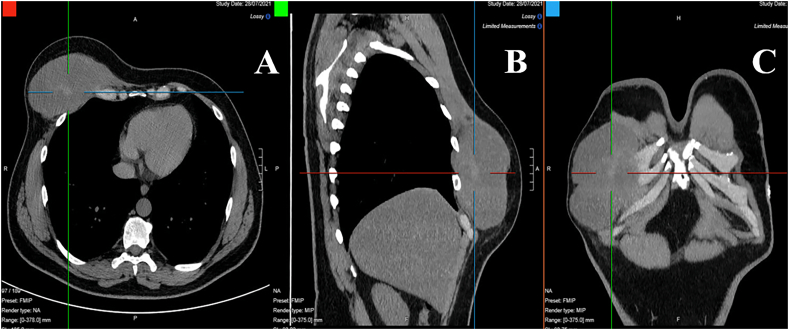
A,B,C. Thoracic contrast-enhanced computed tomography (CECT) showing a smooth contour, heterogeneous and hypodense subcutaneous soft tissue mass, anterior to the right pectoral muscles and to the right 4th–7th rib (panel A axial view, panel B sagittal view, panel C coronal view).

**Fig. 2:**
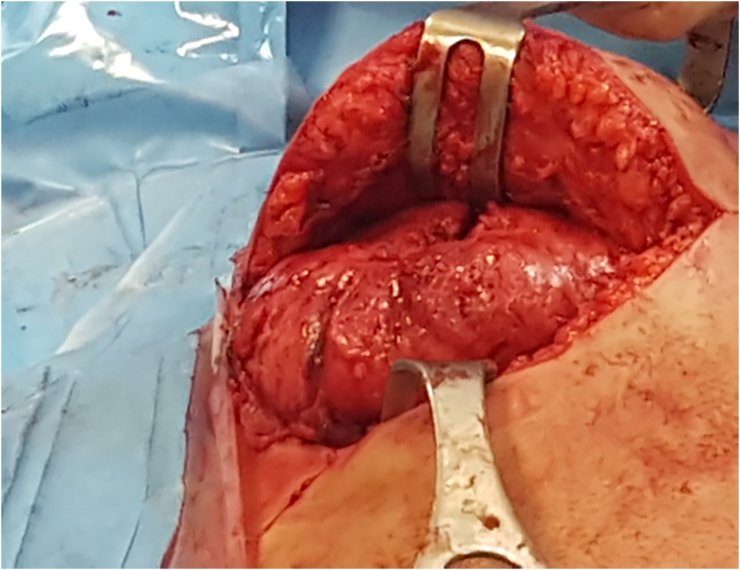
Solid soft tissue tumor tenaciously attached to the underlying muscles and ribs: operative findings.

**Fig. 3:**
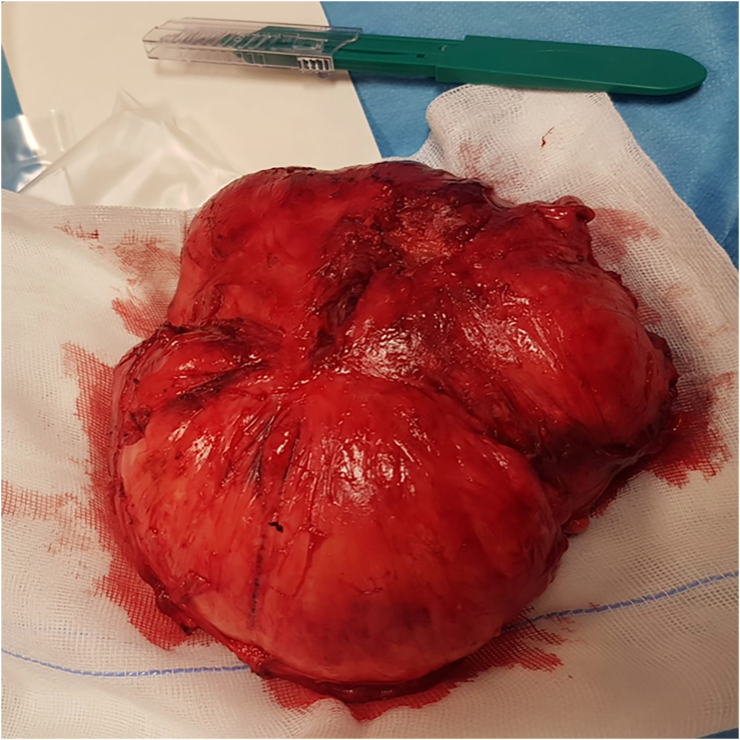
Surgical specimen consisting of a voluminous soft tissue neoplasm of the anterior chest wall measuring 14 × 14 × 6 cm.

**Fig. 4:**
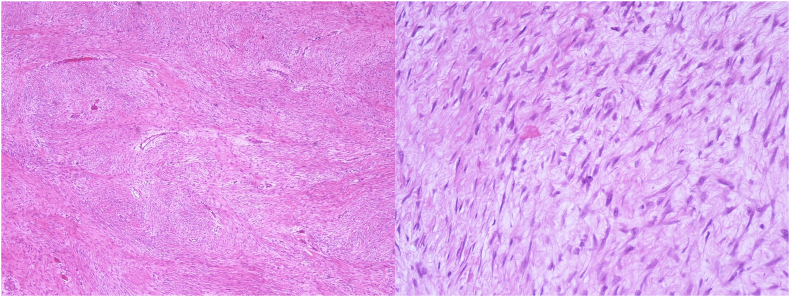
A,B. Spindle cells embedded in a collagenous matrix (panel A haematoxylin and eosin, original magnification x 20, panel B haematoxylin and eosin, original magnification x 40).

**Fig. 5:**
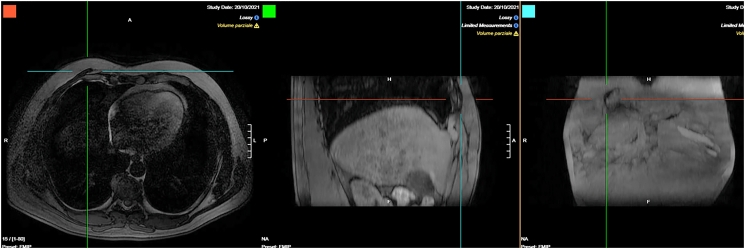
A,B,C. Postoperative thoracic MRI with no evidence of DF local recurrence (panel A axial view, panel B sagittal view, panel C coronal view).

A 43-year-old Caucasian male presented to the Emergency Department with a 6-month history of recurrent painful and enlarging post-traumatic chest wall hematoma. The patient referred a previous blunt thoracic trauma at work 10 months earlier with a hematoma spontaneously resorbed in 2 months. He wasn't taking any drug, referred habit on smoking but denied alcohol consumption; his familial medical history was normal. He was employed, married and of medium socio-economic status. Patient was asymptomatic, his vital signs were normal. Physical examination revealed at the same site of the previous thoracic trauma a partially solid, painful mass of approximately 15 cm on the right anterior chest wall (pectoral region) without swelling of the superficial lymph nodes. Laboratory tests reported neutrophilic leukocytosis. Thoracic contrast-enhanced computed tomography (CECT) showed a smooth contour, heterogeneous and hypodense subcutaneous soft tissue mass (suspicious for an organized hematoma), measuring 13 cm × 6 m, anterior to the right pectoral muscles and to the right 4th–7th rib (Fig. 1). The patient, after understanding his medical condition and accepting surgery, was taken to the operating room for hematoma drainage under general anesthesia. The patient was placed in the supine position on the operating table: intraoperatively we found a solid suprafascial neoplasm (Fig. 2), tenaciously attached to the underlying muscles and ribs, that was completely excised and sent for histological examination (Fig. 3); a subcutaneous right chest drain was placed. The postoperative course was uneventful and the patient was discharged, after the removal of chest drain, on the 2th postoperative day in a stable condition. The surgical specimen consisted of a voluminous solid neoplasm measuring 14 × 14 × 6 cm (Fig. 3). Histopathological examination diagnosed DF of the chest wall with microscopically positive tumor margins (R1 resection). Histopathologically the tissue consisted of spindle cells embedded in a collagenous matrix (Fig. 4) The patient was referred to Oncology Department: oncologists recommended surgery to achieve R0 resection, the patient refused surgical treatment and adjuvant radiation therapy (50 Gy) was performed. The patient, three months after radiotherapy, underwent thoracic magnetic resonance imaging (MRI) without evidence of local recurrence of DF (Fig. 5) and after a follow-up of one year is asymptomatic.

## 3 Discussion

DF is defined as a myofibroblastic proliferative soft tissue tumor and classified as an intermediate malignancy. It is a locally aggressive, nonmetastasizing, well differentiated, unecapsulated neoplasm with a tendency for local invasion and recurrence [5]. DF is more common in patients 15–60 years old with a female predominance [6]. DF can be divided in FAP-related DF (10–15%) and sporadic DF (85–90%) [7]. FAP-related DF is prevalent intra-abdominally and presents up to 10 years earlier than the sporadic DF [8] which develops most commonly in extra-abdominal locations (hip/buttock area, extremities and shoulders) [9]. DF of the chest wall accounts for only 10–20% of all cases [10] with tumor size ranging from 5 to 10 cm and rarely larger than 20 cm [11]. Most thoracic DFs originate from the chest wall, however rare intrathoracic DFs have been described. DF of the chest wall is most commonly found on the anterior chest wall, as in our case, but it can develop on the posterior chest wall and rarely may involve the paraspinous musculature [12]. Factors like trauma, surgery, pregnancy and sex hormones have been associated with the appearance and progression of DF [13]. The central biologic event in the DF formation is an alteration in the Wnt/β-catenin pathway which results in the nuclear accumulation of β-catenin that drives the proliferative process [7]. Diagnosis is difficult and is based on clinical symptoms, physical examination, radiology, biopsy and histopathology. Presentation varies from asymptomatic to disabling tumors; symptoms are related to the site, size, and progression speed. DF of the chest wall commonly presents as a painless slowly enlarging solid mass fixed to deep tissues and often with infiltrative borders; the overlying skin is uninvolved. DF becomes symptomatic due to mechanical compression of neighboring organs; in case of invasion of the nervous structures hyperesthesia, muscular weakness or more rarely pain may develop [11]. There are no specific laboratory tests, radiology is essential for diagnosis, treatment and follow up. DF appearance depends on its composition which can be fibrotic, vascular or cellular according to the stage of the neoplasm. Ultrasound (US) appearance of DF ranges from a smooth contour oval mass to a poorly defined soft tissue neoplasm with variable echogenicity. On CT scan DF varies from well-defined soft tissue mass to infiltrative margins. DF is isodense with the skeletal muscle with areas of hypo or hyper-attenuation according to myxoid or fibrotic elements. Most of DFs bear a moderate contrast enhancement and do not display necrotic areas or calcifications. In our case thoracic CT scan was not diagnostic. MRI is considered the first choice for evaluating the relationship between DF and surrounding structures, such as bone, vessels, nerves. Typical DF shows iso-SI (signal intensities) on T1WI, high SI on T2WI and strong heterogeneous enhancement on enhanced T1WI. The heterogeneous enhancement may correspond to varying proportions of myxoid tissue, cellular tissue, and collagenous stroma on pathology. As it is not possible to distinguish desmoids from sarcomas by imaging, histologic diagnosis is mandatory. Histopathology of DF shows a proliferation of fibroblastic and myofibroblastic-type spindle cells arranged in parallel bundles, with variable cellularity, in the presence of mixoid and edematous areas, coexisting with areas of higher cellular density; atypical nuclei and mitoses are rare [11]. Currently the treatment for DF has shifted from surgery (post-operative recurrence rates of 20%–70%) [14] to conservative therapy including watchful waiting. After diagnosis with US or CT-guided core needle biopsy, “active surveillance” is the first treatment option [15] because of approximately 50% of cases reach a stabilization period and some patients even present regression of DF [16]. Active surveillance includes close monitoring of patients with MRI or CT scan every month for the first two months, then every three months for the first year followed by every six months until the fifth year, and yearly after to detect rapidly progressive cases [13]. Following this approach, only 14–16% of DF will require surgery and a quarter of the patients will show DF regression [13]. In stable DF and in DF that diminish in size, no treatment may be necessary. DF progression during an active surveillance period requires active treatments including surgery, radiotherapy, systemic therapy. Current indications for systemic treatment include rapidly progressive DF or patient rejection to active surveillance. Systemic treatment options include non-steroid anti-inflammatory drugs, anti-hormonal therapies, tyrosine kinase inhibitors and conventional “low dose” chemotherapeutic regimens (doxorubicin, vinblastine and methotrexate) [13], medical therapy requires a period of several months to obtain the anticipated efficacy [17]. Surgery is recommended in cases of DF progression to medical or radiation therapies or if any symptoms appear; it is a potentially curative treatment and can be considered first line if surgical morbidity is limited [6]. Surgery should obtain microscopic negative margins (R0 resection), but R1 resection can be accepted, as in our case, to preserve function. A multicenter Japanese study revealed that R1 resection, recurrent tumor, extremity location and CTNNB1 gene mutation status are adverse prognostic factors for postoperative local recurrence and preoperative drug treatment does not influence local recurrence [18]. Radiotherapy represents an effective and alternative option to surgery and may be the preferred local therapy if there is a high risk of functional consequences following surgery. Long-term local control following definitive RT is achieved in approximately 65–80% of DF [19], similar to large surgical series. Adjuvant radiotherapy (dose of 50–56 Gy), as in our case, could improve local control and reduce recurrence after incomplete resection [20]. Although DF does not metastasize, it can result in significant morbidity and death from loco-regional invasion.

## 4 Conclusion

DF is a rare soft tissue neoplasm that may be locally very aggressive causing significant functional limitations, pain and even major disabilities. The unpredictable course, the low incidence, the lack of knowledge about DF have been limiting factors toward a more rapid advancement of its management. Given the wide spectrum of treatments available for DF there is now the ability to tailor treatment to the patient.

## Sources of funding

All the authors declare that this research didn't receive any specific grant from funding agencies in the public, commercial, or not-for-profit sectors.

## Ethical approval

Ethical approval has been exempted by our institution because this is a case report and no new studies or new techniques were carried out.

## Consent

Written informed consent was obtained from the patient, for publication of this case report and accompanying images. A copy of the written consent is available for review by the Editor-in-Chief of this journal on request.

## Registration of research studies

Not applicable.

## Guarantor

Giuseppe Evola.

## Provenance and peer review

Not commissioned, externally peer-reviewed.

## CRediT authorship contribution statement

Giuseppe Evola: Drafting the manuscript, literature research.

Mario Scravaglieri: Operated on the patient, drafting the manuscript.

Enrico Piazzese: Operated on the patient, drafting the manuscript.

Francesco Roberto Evola: Drafting the manuscript and literature research.

Giovanni Francesco Di Fede: Drafting the manuscript, literatureresearch.

Luigi Piazza: Revising the manuscript.

## Declaration of competing interest

All the authors certify that there is no conflict of interest regarding the material discussed in the manuscript.
